# Supplementary material for: Revised Exon Structure of l-DOPA Decarboxylase (DDC) Reveals Novel Splice Variants Associated with Colorectal Cancer Progression
Source: Int J Mol Sci. 2020 Nov 13;21(22):8568. doi: 10.3390/ijms21228568 (PMC7697000; doi:10.3390/ijms21228568)
Supplement: Supplementary file 1 [file ijms-21-08568-s001.zip › Supplementary Tables/Table S1.docx]

**Table S1.** The panel of 53 human cancerous cell lines used in the current study.

| Type of malignancy | Cultured cell lines |
| --- | --- |
| Breast adenocarcinoma | MCF-7, SK-BR-3, BT-20, MDA-MB-231, MDA-MB-468 |
| Breast ductal carcinoma | BT-474, T-47D, ZR-75-1 |
| Brain cancer | U-87 MG, U-251 MG, D54, H4, SH-SY5Y |
| Colorectal cancer | Caco-2, DLD-1, HT-29, HCT 116, SW 620, COLO 205, RKO |
| Gastric adenocarcinoma | AGS |
| Hepatocellular carcinoma | Hep G2, HuH-7 |
| Leukemia | K-562, HL-60, Jurkat, REC-1, SU-DHL-1, GRANTA-519 |
| Lung adenocarcinoma | A549 |
| Ovarian cancer | OVCAR-3, SK-OV-3, ES-2, MDAH-2774 |
| Prostate cancer | PC-3, DU 145, LNCaP |
| Renal cell carcinoma | ACHN, 786-O, Caki-1 |
| Cervical carcinoma^1^ | HeLa, SiHa |
| Endometrial adenocarcinoma^1^ | Ishikawa, SK-UT-1B |
| Head and neck squamous cell carcinoma^1^ | BB49-SCCHN, CAL-33 |
| Lymphoma^1^ | Raji, Daudi, U-937 |
| Melanoma^1^ | FM3, MDA-MB-435S |
| Urinary bladder cancer^1^ | T24, RT4 |

^1^ *DDC* RNA levels were undetectable in these cancer cell lines; therefore, they were excluded from the succeeding experimental procedure.
