# Supplementary material for: Revised Exon Structure of l-DOPA Decarboxylase (DDC) Reveals Novel Splice Variants Associated with Colorectal Cancer Progression
Source: Int J Mol Sci. 2020 Nov 13;21(22):8568. doi: 10.3390/ijms21228568 (PMC7697000; doi:10.3390/ijms21228568)
Supplement: Supplementary file 1 [file ijms-21-08568-s001.zip › Supplementary Tables/Table S2.docx]

**Table S2.** Primers used in first- and second-round PCRs for the identification of *DDC* novel transcripts in cDNA pools and their semi-quantitative expression analysis in CRC cell lines.

| **Direction** | **Name** | **Sequence (5′→3′)** | **Length (nt)** | **T_m_ (^o^C)** |
| --- | --- | --- | --- | --- |
| **Forward** | L1F | GGAGAATCCCATCAAGGAGAGTAG | 24 | 59 |
|  | N1F | GGACAGAGAGCAAGTCACTCC | 21 | 61 |
|  | 2F | ACGCAAGTGAATTCCGAAGG | 20 | 59 |
|  | 2 new F | CCTCAGGAGCCAGACACGTT | 20 | 63 |
|  | X1F | GGCTCCAACCAGCCTATC | 18 | 59 |
|  | X2F | GCGTCAAGCAGGAGACAG | 18 | 60 |
|  | X3F | TCAAAGTTAGCACGCAGAGCA | 21 | 60 |
|  | X4F | GCAGAAGTTTTAATTACAATGAAGTCCAG | 29 | 57 |
|  | X5F | CAGCCCTGGAGGTGTGT | 17 | 61 |
|  | X9F | TGAGGAAACTGAAGCGGTCA | 20 | 59 |
|  | X6F | GTCACCACACTGCCAATGAA | 20 | 59 |
|  | X8F | CCCAGCACCTGCAACAT | 17 | 58 |
| **Reverse** | X1R | CTCCAGATAGGGTGGGACT | 19 | 59 |
|  | X2R | ACCACTGTGGCAGATCCAT | 19 | 60 |
|  | X3R | CCTGTGGAGATGAACGCAAC | 20 | 60 |
|  | X4R | GACCACACCAAAAACATGATCCA | 23 | 58 |
|  | X5R | CAAGAACCATGCCATGAGGA | 20 | 58 |
|  | X9R | ACGCCGCATTCATTACACTC | 20 | 59 |
|  | X6R | TCGGACGCAAACTCTCACT | 19 | 60 |
|  | X8R | GCTATTCAGACAGCATGTGATCC | 23 | 59 |
|  | 14inR | CAGCTCTTTGATGTGTTCCCAG | 22 | 59 |
|  | 14R | CTACTCCCTCTCTGCTCGCAG | 21 | 63 |
