# Supplementary material for: Revised Exon Structure of l-DOPA Decarboxylase (DDC) Reveals Novel Splice Variants Associated with Colorectal Cancer Progression
Source: Int J Mol Sci. 2020 Nov 13;21(22):8568. doi: 10.3390/ijms21228568 (PMC7697000; doi:10.3390/ijms21228568)
Supplement: Supplementary file 1 [file ijms-21-08568-s001.zip › Supplementary Tables/Table S3.docx]

**Table S3.** Second-round PCR primer pairs, used for the identification of *DDC* novel transcripts in cDNA pools.

| ***DDC* transcript** | **Name of primer** | | **Amplicon size (bp)** |
| --- | --- | --- | --- |
|  | **Forward** | **Reverse** |  |
| **v.32, v.33** | 2F | X1R | 434 |
| **v.34** |  |  | 320 |
| **v.32, v.33, v.34** | X1F | 14inR | 1200 |
| **v.36** | 2F | X2R | 689 |
| **v.37** |  |  | 575 |
| **v.36, v.37** | X2F | 14inR | 982 |
| **v.38, v.39** | 2F | X3R | 883 |
| **v.40, v.41** |  |  | 769 |
| **v.38, v.39, v.40, v.41** | X3F | 14inR | 840 |
| **v.48** | 2F | X4R | 783 |
| **v.49** |  |  | 669 |
| **v.48, v.49** | X4F | 14inR | 794 |
| **v.42** | 2F | X5R | 790 |
| **v.43** |  |  | 676 |
| **v.42, v.43** | X5F | 14inR | 764 |
| **v.30** | 2F | X9R | 1041 |
| **v.31** |  |  | 927 |
| **v.30, v.31** | X9F | 14inR | 590 |
| **v.44, v.35** | 2F | X6R | 1076 |
| **v.45** |  |  | 962 |
| **v.44, v.35, v.45** | X6F | 14inR | 577 |
| **v.46** | 2F | X8R | 1290 |
| **v.50** |  |  | 1176 |
| **v.46, v.50** | X8F | 14inR | 418 |
