# Supplementary material for: Revised Exon Structure of l-DOPA Decarboxylase (DDC) Reveals Novel Splice Variants Associated with Colorectal Cancer Progression
Source: Int J Mol Sci. 2020 Nov 13;21(22):8568. doi: 10.3390/ijms21228568 (PMC7697000; doi:10.3390/ijms21228568)
Supplement: Supplementary file 1 [file ijms-21-08568-s001.zip › Supplementary Tables/Table S4.docx]

**Table S4.** Primers used in PCR-based pre-amplification and real-time PCR for the quantification of the *DDC* novel transcripts bearing one of exons X1, X3, X8, and X9, relatively to *HPRT1* mRNA expression.

|  | **Direction** | **Name** | **Sequence (5′→3′)** | **Length (nt)** | **T_m_ (^o^C)** |
| --- | --- | --- | --- | --- | --- |
| **PCR-based pre-amplification** | **Forward** | HPRT1 F | AACCTCTCGGCTTTCCCG | 18 | 61 |
|  |  | 2F | ACGCAAGTGAATTCCGAAGG | 20 | 59 |
|  |  | 5F | ACCAAAGTGATCCATCGGCT | 20 | 60 |
|  |  | 8F | AGTTCCGGCACCTTCTGAAT | 20 | 60 |
|  |  | 12F | ACTGGGCAGAAGATTTCGCT | 20 | 60 |
|  | **Reverse** | HPRT1 R | CAGTGCTTTGATGTAATCCAGCAG | 24 | 59 |
|  |  | 4R | TCTTCCCGAGCCAGTCCAT | 19 | 62 |
|  |  | 7R | GTCAAAGGAGCAGCATGTTGTG | 22 | 60 |
|  |  | 10R | CTGAATCCTGATGGCTGTGCT | 21 | 61 |
|  |  | 13R | GAAAGCAGACAAGCCCCAGA | 20 | 61 |
| **Real-time PCR** | **Forward** | HPRT1 2F | TGGAAAGGGTGTTTATTCCTCAT | 23 | 57 |
|  |  | X1 new F | AGAGGCTCCAACCAGCCTA | 19 | 59 |
|  |  | X3F | TCAAAGTTAGCACGCAGAGCA | 21 | 60 |
|  |  | X9F | TGAGGAAACTGAAGCGGTCA | 20 | 59 |
|  |  | X8 new F | TAGAATGAGCAAGGCACGGG | 20 | 60 |
|  | **Reverse** | HPRT1 3R | ATGTAATCCAGCAGGTCAGCAA | 22 | 60 |
|  |  | X1 new R | GCTAGGCCCTTTTCCAGAGA | 20 | 59 |
|  |  | X3R | CCTGTGGAGATGAACGCAAC | 20 | 60 |
|  |  | X9R | ACGCCGCATTCATTACACTC | 20 | 59 |
|  |  | X8R | GCTATTCAGACAGCATGTGATCC | 23 | 59 |
