# Supplementary material for: Revised Exon Structure of l-DOPA Decarboxylase (DDC) Reveals Novel Splice Variants Associated with Colorectal Cancer Progression
Source: Int J Mol Sci. 2020 Nov 13;21(22):8568. doi: 10.3390/ijms21228568 (PMC7697000; doi:10.3390/ijms21228568)
Supplement: Supplementary file 1 [file ijms-21-08568-s001.zip › Supplementary Tables/Table S5.docx]

**Table S5.** Pre-amplification and real-time PCR primer pairs, used for the quantification of the *DDC* novel transcripts bearing one of exons X1, X3, X8, and X9, relatively to *HPRT1* mRNA expression.

| **Gene** | **Transcript** | **Name of primer** | | **Amplicon size (bp)** |
| --- | --- | --- | --- | --- |
|  |  | **Forward** | **Reverse** |  |
| ***HPRT1*** | **(unique)** | HPRT1 F | HPRT1 R | 370 |
|  | **(unique)** | HPRT1 2F | HPRT1 3R | 151 |
| ***DDC*** | **v.32, v.33** | 2F | 4R | 492 |
|  | **v.34** |  |  | 378 |
|  | **v.32, v.33, v.34** | X1 new F | X1 new R | 71 |
|  | **v.38, v.39, v.40, v.41** | 5F | 7R | 457 |
|  |  | X3F | X3R | 136 |
|  | **v.30, v.31** | 8F | 10R | 299 |
|  |  | X9F | X9R | 100 |
|  | **v.46, v.50** | 12F | 13R | 368 |
|  |  | X8 new F | X8R | 71 |
