# Supplementary figures and images for: Revised Exon Structure of l-DOPA Decarboxylase (DDC) Reveals Novel Splice Variants Associated with Colorectal Cancer Progression
Source: Int J Mol Sci. 2020 Nov 13;21(22):8568. doi: 10.3390/ijms21228568 (PMC7697000; doi:10.3390/ijms21228568)

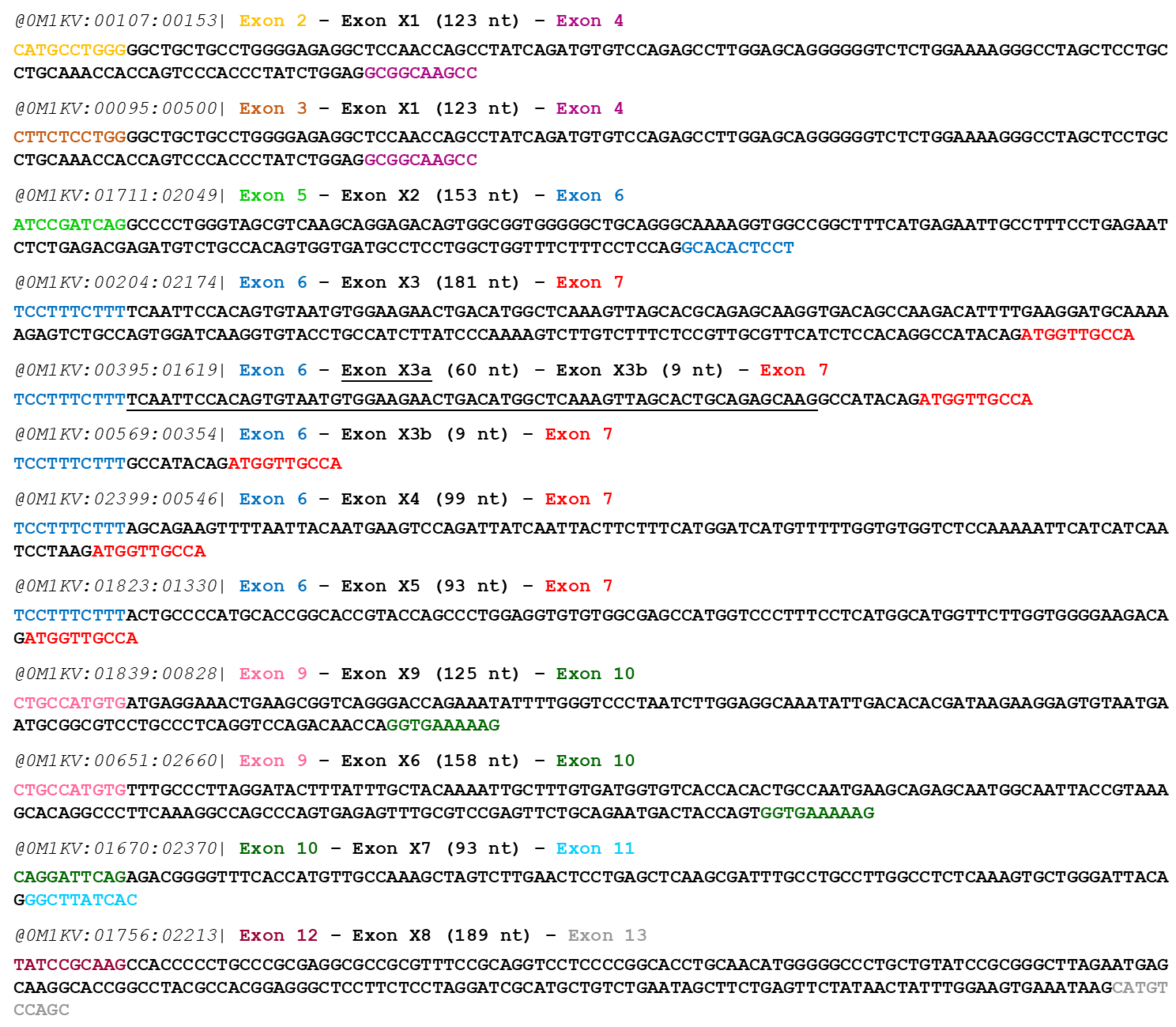

Supplement: Supplementary file 1 [file ijms-21-08568-s001.zip › Supplementary Figures/Figure S1.tif]

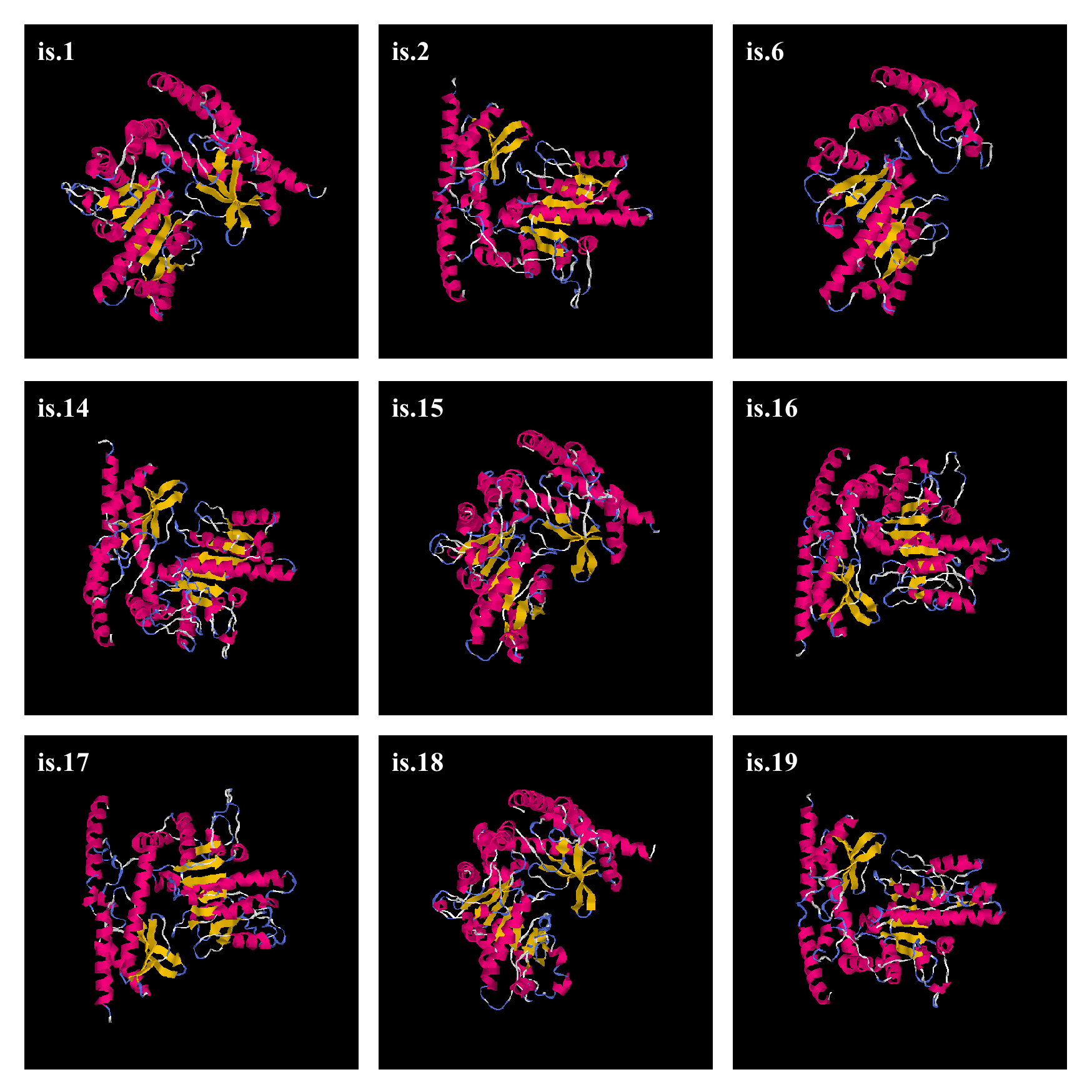

Supplement: Supplementary file 1 [file ijms-21-08568-s001.zip › Supplementary Figures/Figure S2.tif]

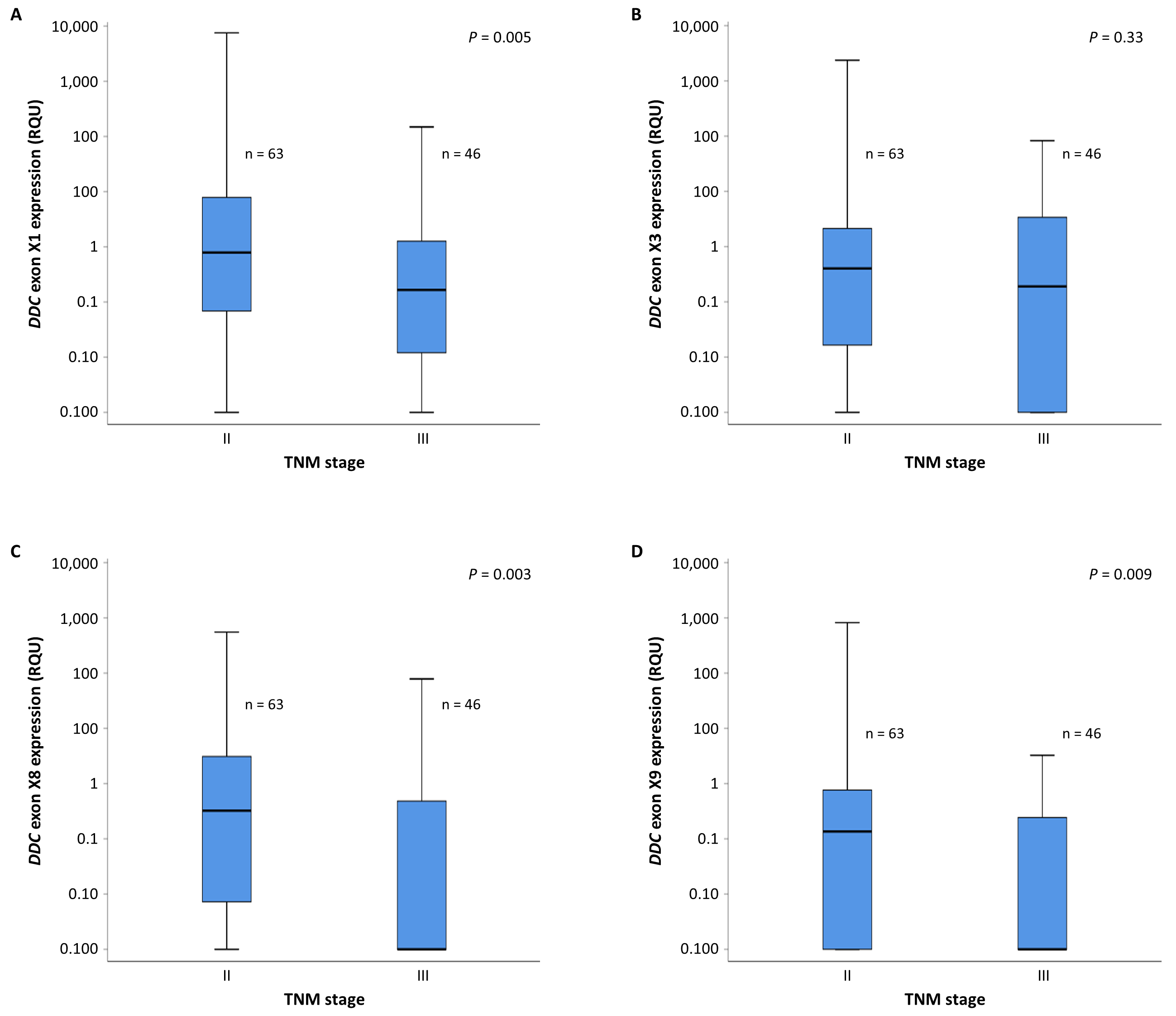

Supplement: Supplementary file 1 [file ijms-21-08568-s001.zip › Supplementary Figures/Figure S3.tif]

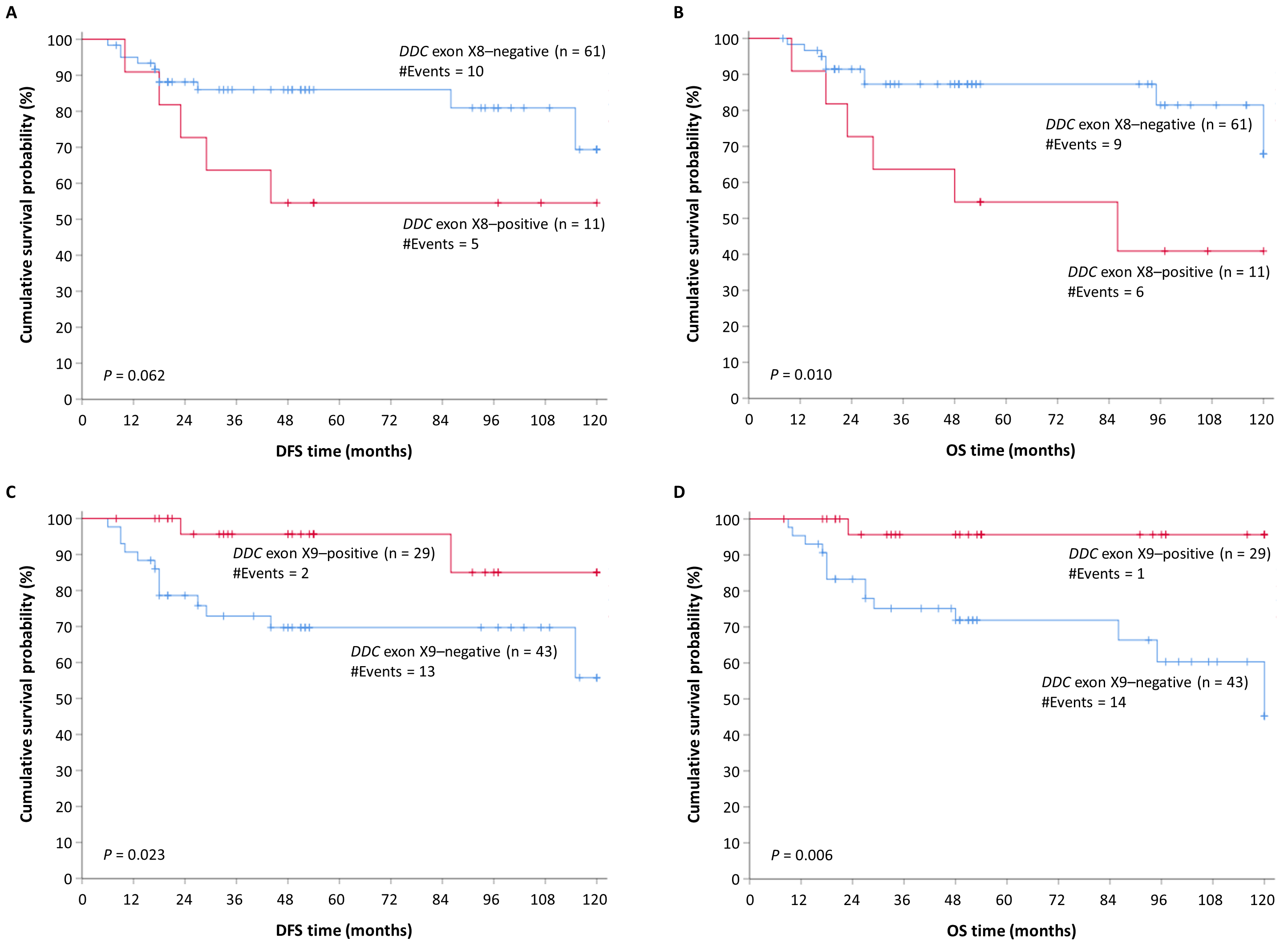

Supplement: Supplementary file 1 [file ijms-21-08568-s001.zip › Supplementary Figures/Figure S4.tif]

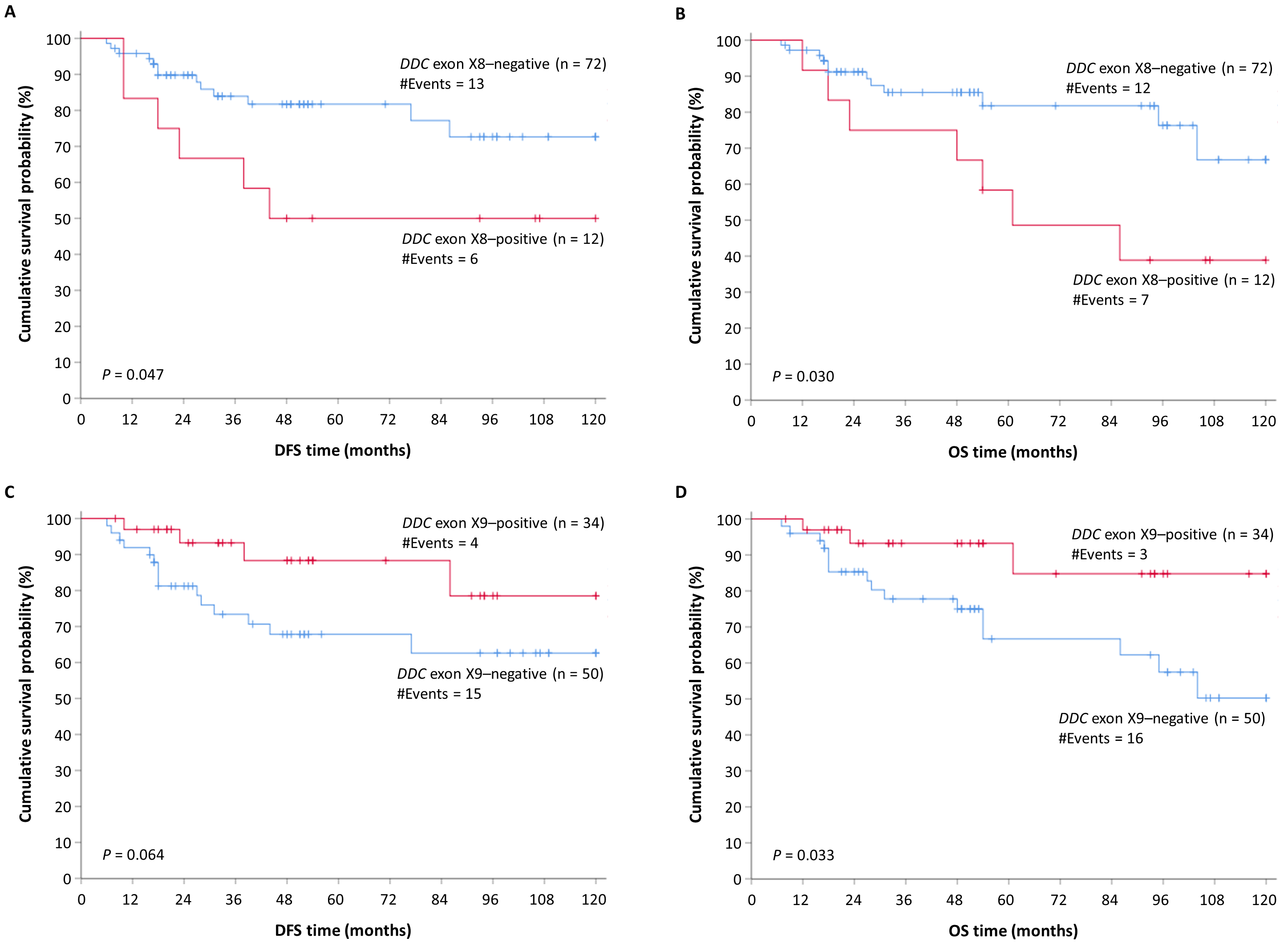

Supplement: Supplementary file 1 [file ijms-21-08568-s001.zip › Supplementary Figures/Figure S5.tif]

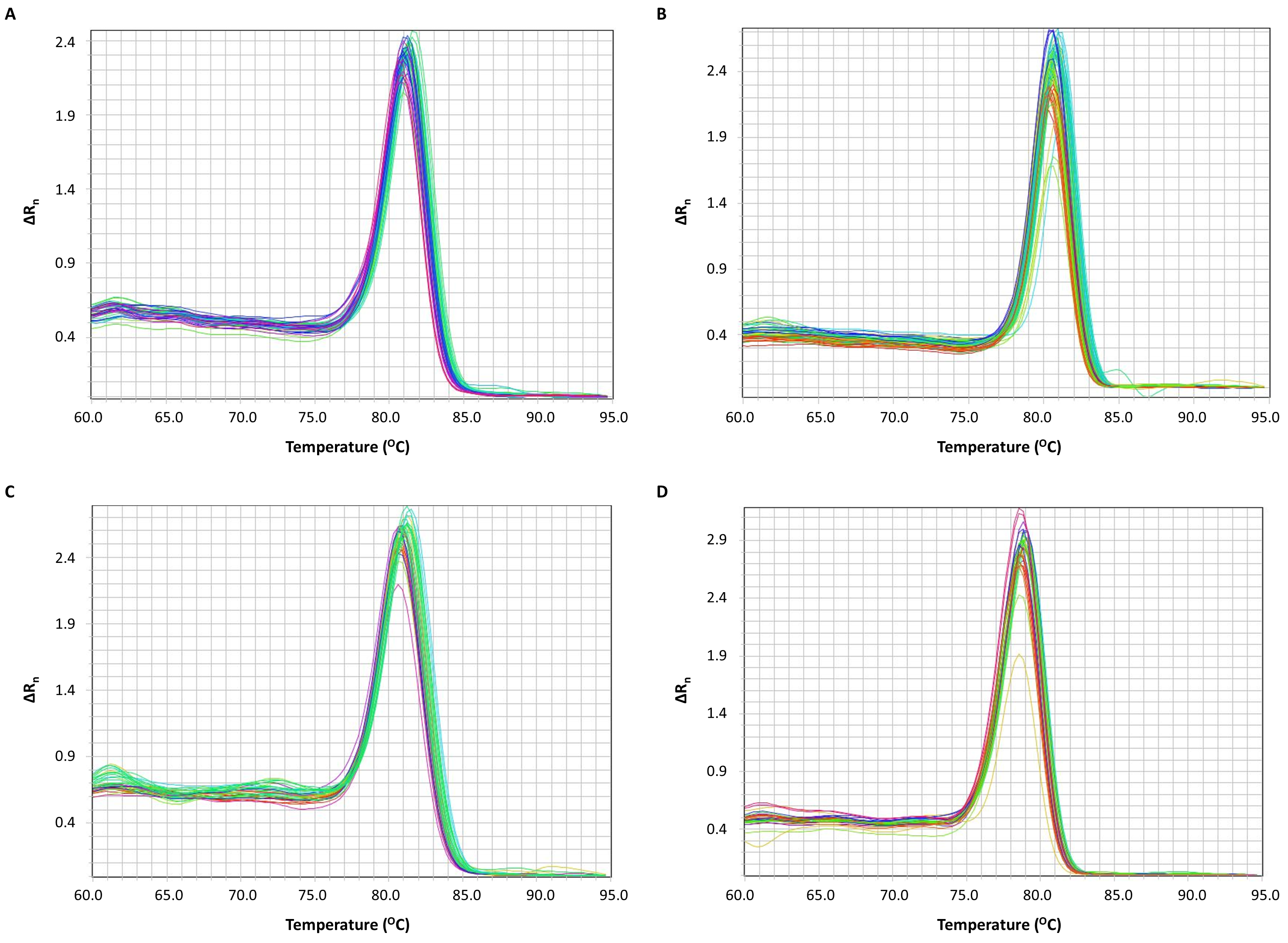

Supplement: Supplementary file 1 [file ijms-21-08568-s001.zip › Supplementary Figures/Figure S6.tif]

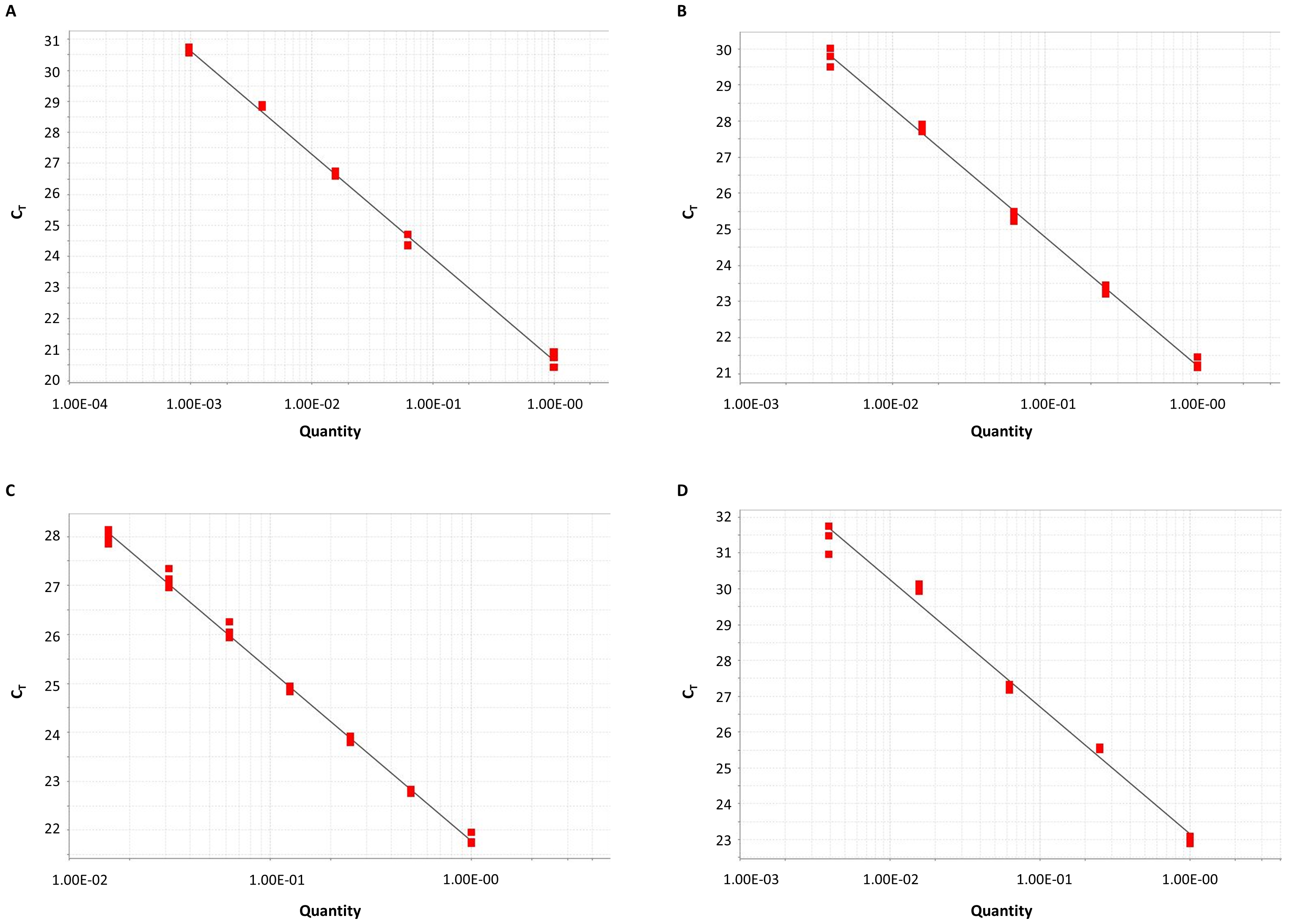

Supplement: Supplementary file 1 [file ijms-21-08568-s001.zip › Supplementary Figures/Figure S7.tif]
